# Supplementary material for: The Impact of Physical Activity on Mental Health during COVID-19 Pandemic in China: A Systematic Review
Source: Int J Environ Res Public Health. 2022 May 28;19(11):6584. doi: 10.3390/ijerph19116584 (PMC9180501; doi:10.3390/ijerph19116584)
Supplement: Supplementary file 1 [file ijerph-19-06584-s001.zip › ijerph-1670195-supplementary.pdf]

Table S1. Study quality assessment.

Criterion \ Study ID	1	2	3	4	5	6	7	8	9	10	11	12	13	14	15	16	17	18	19	20	21	22	23
1. Was the research question or objective in this paper clearly stated?	1	1	1	1	1	1	1	1	1	1	1	1	1	1	1	1	1	1	1	1	1	1	1
2. Was the study population clearly specified and defined?	1	1	1	1	1	1	1	1	1	1	1	1	1	1	1	1	1	1	1	1	1	1	1
3. Was the participation rate of eligible persons at least 50%?	1	1	1	1	1	1	1	1	1	1	1	1	1	1	1	1	1	1	1	1	1	1	1
4. Were all the subjects selected or recruited from the same or similar populations (including the same time period)? Were inclusion and exclusion criteria for being in the study pre-specified and applied uniformly to all participants?	1	1	1	1	1	1	1	1	1	1	1	1	1	1	1	1	1	1	1	1	1	1	1
5. Was sample size justification, power description, or variance and effect estimates provided?	0	0	0	0	0	0	0	0	1	0	0	0	0	0	0	0	0	0	1	0	0	0	0
6. For the analyses in this paper, were the exposure(s) of interest measured prior to the outcome(s) being measured?	0	0	0	0	1	0	0	0	0	0	0	0	0	0	0	0	0	0	0	0	0	0	0
7. Was the timeframe sufficient so that one could reasonably expect to see an association between exposure and outcome if it existed?	0	0	0	0	0	0	0	0	0	0	0	0	0	0	0	1	0	0	0	0	0	0	0
8. For exposures that can vary in amount or level, did the study examine different levels of the exposure as related to the outcome (e.g., categories of exposure, or exposure measured as continuous variable)?	0	1	1	0	0	1	1	1	1	0	1	1	1	0	1	1	0	1	1	1	1	1	0
9. Were the exposure measures (independent variables) clearly defined, valid, reliable, and implemented consistently across all study participants?	0	0	0	0	1	1	0	1	1	0	1	1	1	0	1	1	0	0	0	1	1	0	1
10. Was the exposure(s) assessed more than once over time?	0	0	0	0	0	0	0	0	0	0	0	0	0	0	0	1	0	0	0	0	0	0	0
11. Were the outcome measures (dependent variables) clearly defined, valid, reliable, and implemented consistently across all study participants?	0	1	0	1	1	1	1	1	1	0	1	0	1	0	1	1	1	1	1	1	1	1	1
12. Were the outcome assessors blinded to the exposure status of participants?	0	0	0	0	0	0	0	0	0	0	0	0	0	0	0	0	0	0	0	0	0	0	0
13. Was loss to follow-up after baseline 20% or less?	0	0	0	0	0	0	0	0	0	0	0	0	0	0	0	1	0	0	0	0	0	0	0
14. Were key potential confounding variables measured and adjusted statistically for their impact on the relationship between exposure(s) and outcome(s)?	0	0	0	0	1	0	0	1	1	1	0	0	1	0	1	1	0	0	0	1	1	0	0
**Total score**	4	6	5	5	8	7	6	8	9	5	7	6	8	4	8	11	5	6	7	8	8	6	6

Notes: This study quality assessment tool was adopted from the National Institutes of Health's Quality Assessment Tool for Observational Cohort and Cross-Sectional Studies. For each criterion, a score of one was assigned if "Y" was the response, whereas a score of zero was assigned otherwise. A study-specific global score, ranging from 0 to 14, was calculated by summing up scores across all 14 criteria. Study quality assessment helped measure strength of scientific evidence, but was not used to determine the inclusion of studies.
